# Plasma Concentrations of Selected Organobromine Compounds and Polychlorinated Biphenyls in Postmenopausal Women of Québec, Canada

**DOI:** 10.1289/ehp.10303

**Published:** 2007-07-24

**Authors:** Torkjel M. Sandanger, Marc Sinotte, Pierre Dumas, Mario Marchand, Courtney D. Sandau, Daria Pereg, Sylvie Bérubé, Jacques Brisson, Pierre Ayotte

**Affiliations:** 1 Unité de recherche en santé publique, Centre hospitalier universitaire de Québec and Université Laval, Québec, Québec, Canada; 2 Norwegian Institute for Air Research, Polar Environmental Centre, Tromsø, Norway; 3 Unité de recherche en santé des populations, Centre Hospitalier Affilié Universitaire de Québec, Québec, Québec, Canada; 4 Centre de Toxicologie, Institut national de santé publique du Québec, Québec, Québec, Canada; 5 Trium Environmental Solutions Inc., Cochrane, Alberta, Canada

**Keywords:** brominated flame retardants, polybrominated biphenyls, polybrominated diphenyl ethers, polychlorinated biphenyls, postmenopausal women, Québec, Canada

## Abstract

**Background:**

Brominated flame retardants, especially polybrominated diphenyl ethers (PBDEs), have been widely used in North America, but little is known about the level of exposure of human populations to these compounds.

**Objectives:**

We set out to assess the internal exposure of postmenopausal Canadian women to selected organobromine compounds and to investigate factors associated with this exposure.

**Methods:**

We measured concentrations of four PBDEs, one polybrominated biphenyl, and for comparative purposes, 41 polychlorinated biphenyl (PCB) congeners in plasma samples from 110 healthy postmenopausal women who were recruited at a mammography clinic in 2003–2004.

**Results:**

PBDE-47 was the major PBDE congener, with a mean (geometric) concentration of 8.1 ng/g lipids and extreme values reaching 1,780 ng/g. By comparison, the mean concentration of the major PCB congener (PCB-153) was 41.7 ng/g and the highest value was 177 ng/g. PBDEs 47, 99, and 100 were strongly intercorrelated, but weaker correlations were noted with PBDE-153. As the sum of PBDEs (∑PBDEs) increased, the relative contribution of PBDE-47 to the ∑PBDEs increased, whereas that of PBDE-153 decreased. PBDE-153 was the only brominated compound correlated to PCB-153. PBDE levels were not linked to any sociodemographic, anthropometric, reproductive, or lifestyle variables documented in the present study. Age and body mass index gain since the age of 18 years were significant predictors of PCB-153 plasma levels.

**Conclusion:**

Our results suggest that exposure to PBDE-47 likely occurs through direct contact with the penta-PBDE formulation, whereas exposure to PBDE-153 may originate in part from the food chain.

Organobromine compounds such as polybrominated diphenyl ethers (PBDEs) are now largely distributed in the environment because of their wide use as flame retardants in electronic equipment, plastics, and textiles. Several PBDE congeners, their hydroxylated metabolites, and brominated bisphenol A analogs induce the estrogen-receptor signal transduction pathway *in vitro* and may therefore increase the risk of hormone-related diseases (Meerts et al. 2001). Studies in Sweden have revealed that PBDEs increased in human breast milk over the last decades (Meironyté et al. 1999; Norén and Meironyté 2000). However, in recent years a decrease in concentrations has been noted in Sweden (Lind et al. 2003). A similar decline in levels has not been reported in plasma samples from people in North America; to the contrary, limited data for the U.S. population indicate a steep increase in PBDE blood concentrations between 1973 and 2003 (Schecter et al. 2005b). The human levels in North America are also, in general, considerably higher than in any other parts of the world.

In the course of a pilot study investigating possible environmental risk factors of breast cancer, we analyzed plasma samples obtained from 110 healthy postmenopausal women living in the Québec City area (Québec, Canada) for several persistent organic pollutants. In this article we present the concentrations of 4 PBDE, 1 polybrominated biphenyl (PBB), and 41 polychlorinated biphenyl (PCB) congeners, the latter for comparative purposes. We also investigated the relation between concentrations of these compounds and anthropometric, demographic, reproductive, and lifestyle characteristics of participants.

## Material and Methods

### Population

We recruited a convenience sample of 110 women at a large mammography screening clinic located in Québec City. All women attending the clinic between July 2003 and March 2004 were given a pamphlet describing the purpose of the study and a form to complete (name and coordinates) if they were interested in participating. A research nurse later contacted by telephone each woman who showed interest to verify her eligibility and schedule an appointment for a face-to-face interview. Women were eligible if *a*) they were postmenopausal; *b*) they had no history of breast cancer; *c*) they had no history of health problems related to steroid hormone metabolism, hepatic, thyroid, or adrenal disease; and *d*) they had not taken hormone replacement therapy during the last 3 months. Women agreeing to participate provided informed consent including authorization for blood sampling and banking. The protocol of the study was approved by the Research Ethics Review Board of Hôpital St-Sacrement – Centre Hospitalier Affilié Universitaire de Québec.

During the face-to-face interview, the research nurse documented participant gynecologic and reproductive histories, current diseases and drug intake, lifestyle habits, and the consumption of some food items including meat. Women were queried about their weight and height at 18 years of age. The research nurse also recorded current anthropometric measures (weight, height, waist circumference) and collected blood samples (75 mL) for various biomarker measurements. Blood specimens were collected in vacutainers with EDTA as the anticoagulant and were kept on ice until transported by medical courier to the laboratory at the end of each morning and afternoon. Blood was processed within 2–3 hr of collection. Samples were centrifuged and the plasma aliquoted and stored at −80°C in glass vials (prewashed with hexane) until analysis.

### Analytical procedure

Plasma samples were extracted on an Oasis HLB (540 mg; Waters Corp., Milford, MA, USA) solid phase extraction (SPE) column according to the method presented by Sandau et al. (2003). Internal standards were added to 5 mL of plasma prior to formic acid (5 mL) and deionized water (5 mL). The mixture was vortexed for 1 min and left overnight in the refrigerator. The HLB column was conditioned with dichloromethane (5 mL), followed by 5% methanol in 0.1 M hydrochloric acid (5 mL). The sample was then slowly applied to the column at a flow rate of 0.38 mL/min. After drying the column with pressurized nitrogen, the sample was extracted using 10% methanol in dichloromethane (15 mL). The sample was evaporated to dryness to ensure that all methanol was removed from the sample before it was redissolved in 0.5 mL *n*-hexane. The extract was subsequently eluted through a column containing 1 g activated Florisil (60–100 mesh; Fisher Scientific, Pittsburgh, PA, USA). The fraction containing the PBDEs was eluted using hexane/dichloromethane (9/1; 9 mL). The extraction and cleanup procedures were automated using a Rapidtrace Automated SPE workstation (Zymark Corp., Hopkinton, MA, USA), and evaporation was performed using a heated vacuum evaporator (Rapidvap; Labconco Corp., Kansas City, MO, USA). ^13^C_12_-PBDE-77 was used as the internal standard for the PBDEs, and four different ^13^C_12_-PCBs (PCBs 77, 101, 141, and 178) were used as internal standards for the PCBs (Cambridge Isotope Laboratories, Andover, MA, USA).

Samples were analyzed on an Agilent 6890N gas chromatograph equipped with split/splitless injector, Agilent 7683 auto-sampler, Agilent DB-XLB column (60 m; 0.25 mm i.d., 0.25 μm film thickness), coupled with an Agilent 5973N mass spectrometer (Agilent Technologies, Wilmington, DE, USA). The carrier gas (helium) flow was 0.8 mL/min (flow controlled). The temperature program was as follows: 100°C (1 min), 10°C/min to 200°C (0 min), 1.5°C/min to 240°C (10 min), 20°C/min to 330°C (10 min). The injector and transfer lines were kept at 270°C and 280°C, respectively. The injection volume was 2 μL, injected in a pulsed splitless mode.

The mass spectrometer was operated in selected ion monitoring mode, using electron capture negative ionization with methane (99.97% purity) as the reagent gas. The ion source and quadropole temperatures were set to 150°C and 103°C, respectively. Masses 79 and 81 were monitored for all brominated compounds. For PCBs, the molecular ion and M-2 fragment ion were monitored as the target and confirmation ions. The target ion was employed for quantification, and the confirmation ion from the same isotopic cluster was used to confirm the identity of the compound.

We determined concentrations of total cholesterol, free cholesterol, triglycerides, and phospholipids in plasma samples by enzymatic methods. The total plasma lipid concentration was calculated according to the equation proposed by Akins et al. (1989).

### Quality assurance/quality control

The accuracy of the analytical method was assured through analysis of certified reference materials and participation in international interlaboratory comparison programs both for PCBs and PBDEs. In addition, the precision was monitored by analyzing 15 samples from a control serum pool. The analysis of the serum pool samples gave reproducible results for the PBDEs. For PBDE-47, the mean concentration of the control pool samples was 422 ng/L and the coefficient of variation (CV) was 11%. For comparison, the mean PCB-153 concentration was 328 ng/L and the CV was 6%. The accuracy of the PBDE and PCB analyses was assured through the quantification of the NIST SRM (standard reference material) 1589a (National Institute of Standards and Technology, Gaithersburg, MD, USA). The mean concentration of PBDE-47 measured in this reference material was 193 ng/L, a value 12% above the mean reference value of 172 ng/L (range, 162–182 ng/L). For PBDE-99, we obtained a mean value of 48 ng/L, which is 20% above the reference value of 39.9 ng/L (range, 34.7–45.1 ng/L). For PBDE-100 we obtained the same value as the reference value (i.e., 25 ng/L). For PCB-153, the mean concentration was 833 ng/L, which is 11% below the reference value of 936 ng/L (range, 891–981 ng/L). The average concentration of PBDE-47 in blank samples was 16 ng/L (SD, 5 ng/L). For PBDEs 99 and 100, the blank values were 10 and 2 ng/L, respectively. PBDE-153, PBB-153, and PCB-153 were not detected in blanks. The blank values were subtracted from all concentrations before the results were reported. The recovery rates for ^13^C_12_-PBDE-77 were between 40 and 90%. The recovery rates for ^13^C_12_-PCBs were between 50 and 95%. The limit of detection (LOD) was 4 ng/L for PBDE-47 and 2 ng/L for the other PBDE congeners and PBB-153. The LOD ranged from 3 to 5 ng/L for PCB congeners.

### Statistics

We used the SPSS software package (v. 11.0; SPSS, Chicago, IL, USA) to perform all statistical analyses. PBDEs and PCBs plasma concentrations were log-transformed before correlation and multiple linear regression analyses because the distributions of values were skewed to the right. We performed multiple linear regression analyses to determine predictors of PBDE and PCB plasma levels. Independent variables of interest for these analyses were age, body mass index (BMI), BMI gain since 18 years of age, parity and breast-feeding history, meat consumption, alcohol consumption, and smoking habits. Results from multiple linear regressions were analyzed for the detection of multi-colinearity problems. Statistical tests were two-sided, and a confidence level of 0.05 was used as the criteria for statistical significance.

## Results

The characteristics of participating women are shown in Table 1. The mean age of the 110 women was 58.3 years (range, 48–76 years). All were Caucasian except for one woman of Mediterranean origin. Twenty-five percent of the women were nulliparous, and 13% had had ≥ 4 children. Of the 83 parous women, 40 (48.2%) never breast-fed. Only 7.3% of participants were current smokers, and they smoked an average of 12 cigarettes/ day (range, 3–30). The vast majority (93.6%) of women had consumed alcohol during the year before the interview, and 30% had done so the day before the interview. Mean alcohol intake was one-half of a drink per day, ranging from 0 to 3 drinks/day. More than two-thirds of women had reached the college or university level of education. In general, women with more education tended to consume more alcohol, to breast-feed more, and had a lower BMI.

The concentrations of the four PBDE congeners and PBB-153 are shown in Table 2. PBDE-47 was the congener with the highest geometric mean (GM) concentration (8.1 ng/g lipids) and was detected in all samples. It was the major congener in 96.4% of the participants (106/110). PBDEs 99, 100, and 153 were also frequently detected (≥ 83% of samples), but they were present in much lower concentrations (GM concentrations ranging from 1.1 to 1.4 ng/g lipids). PBDE-153 was the dominant congener in 3.6% of the participating women (4/110). PBB-153 was detected in only 30% of the samples, at levels considerably lower than those of the PBDEs. The arithmetic means of untransformed values were considerably higher than the median values for the PBDEs due to three women with very high levels. The maximum value observed for PBDE-47 was 1,780 ng/g lipids (Table 2). This concentration is 10 times higher than the highest value observed for PCB-153 (177 ng/g lipids), which is the most abundant PCB congener (Table 3). Several other brominated compounds were also detected in the samples with elevated values. These were not identified due to lack of standards.

As mentioned above, distributions of PBDE and PCB congener concentrations were skewed right and values were log-transformed prior to performing statistical analyses. Three women were still considered outliers on the basis of their log-transformed ∑PBDE (sum of PBDEs) concentrations that exceeded the mean value plus 4 SDs. These women were removed before performing additional statistical analyses that aimed at identifying factors associated with PBDE concentrations.

**Figure 1:**
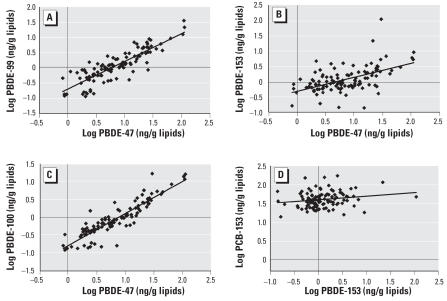
Correlations between plasma concentrations of PBDE-47 and PBDE-99 (*A;* Pearson’s *r* = 0.86; *p* < 0.001), PBDE-153 (*B;* Pearson’s *r* = 0.53; *p* < 0.001), and PBDE-100 (*C*; Pearson’s *r* = 0.90; *p* < 0.001; *n* = 110 for each) and between PBDE-153 and PCB-153 (*D;* Pearson’s *r* = 0.19; *p* < 0.046*; n* = 109) in postmenopausal women from Québec, Canada.

**Figure 2:**
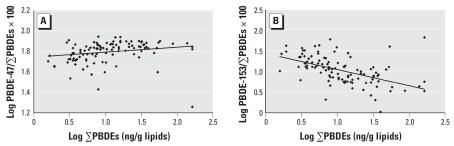
Correlations between plasma concentrations of the ∑PBDEs and relative contributions of PBDE-47 (*A;* Pearson’s *r* = 0.22; *p* < 0.026) and PBDE-153 (*B;* Pearson’s *r* = 0.49; *p* < 0.001) to the ∑PBDE levels in 110 postmenopausal women from Québec, Canada.

PBDEs 47, 99, and 100 were highly inter-correlated (Pearson’s *r* > 0.86; *p* < 0.001; Figure 1A, B). PBDE-153 behaved differently from the other PBDEs, as it was not as highly correlated to the other congeners; for example, the correlation coefficient between PBDE-153 and PBDE-47 was 0.53 (*p* < 0.001; Figure 1C). PBDEs 47, 99, and 100 were not correlated to PCB-153. Only PBDE-153 was weakly correlated to PCB-153 (*r* = 0.19; *p* = 0.046; Figure 1D). The relative contribution of PBDE-47 to the ∑PBDEs increased with the increasing ∑PBDEs (Figure 2A), as opposed to PBDE-153, which represented a decreasing proportion of the ∑PBDEs as the ∑PBDEs increased (Figure 2B).

The lipid-adjusted concentrations of PCB congeners that were detected in ≥ 50% of the samples are presented in Table 3. Congeners that were detected in < 50% of the samples were PCBs 28, 44, 49, 52, 70, 82, 87, 101, 110, 128, 151, 158, 169, 171, 191, 205, and 208. These congeners are not discussed further and are not included in ∑PCB values. The dominating PCB congener was PCB-153, with a GM concentration of 41.7 ng/g lipids (range, 14.4–177), followed by PCBs 180, 138, 163, and 118. The GM ∑PCB level was 204 ng/g lipids (range, 93.1–1,010). Concentrations of the major PCB congeners and of total PCBs were highly intercorrelated (data not shown), and the major congener, PCB-153, was used in the statistical analyses as a surrogate of PCB exposure.

We tested age, BMI, BMI gain since the age of 18 years, parity, cumulative breast-feeding duration, meat consumption, alcohol consumption, and smoking habits for associations with levels of PBDEs and PCBs (PCB-153 as the surrogate). None of these factors was related to the plasma concentrations of PBDEs. Current BMI and BMI gain since 18 years of age were negatively correlated to PCB-153 plasma concentrations (current BMI: *r* = −0.40, *p* < 0.001; BMI gain: *r* = −0.54, *p* < 0.001), whereas age was positively related to PCB-153 concentrations (*r* = 0.23, *p* = 0.015). Multiple linear regression analyses revealed that PCB-153 levels were positively associated with age (*p* = 0.01) and negatively associated with BMI gain (*p* < 0.001). Current BMI was also positively related to PCB-153 concentrations in the final model, although the association did not reach statistical significance (*p* = 0.06). The total model explained 34% of the variance in PCB-153 concentrations (Table 4).

## Discussion

We analyzed plasma samples collected from postmenopausal women residing in the Québec City area for PBDEs, which along with other brominated flame retardants are emerging as contaminants of interest because of their persistence and toxicologic properties similar to those of PCBs. Although mean concentrations of PBDE congeners were much lower than those of major PCB congeners, a few women displayed high PBDE concentrations that exceeded those of major PCBs by an order of magnitude, suggesting that sources of direct exposure are still present in the home and the workplace (Birnbaum and Hubal 2006).

Comparative data from studies conducted in the United States and Europe are presented in Table 5. For these comparisons we have not distinguished between different age groups or sex because there have been no previous reports of levels of PBDEs increasing with age or being different in men and women (Birnbaum and Hubal 2006). Because PBDEs are lipophilic compounds, we present data on a lipid basis to ensure their comparability, whatever the biological sample analyzed (whole blood, serum, plasma, or adipose tissue). From the data presented in Table 5, it is clear that the levels of PBDEs in the women from Québec are substantially (up to 10-fold) higher than the levels in Europe. The levels of PBDEs in our sample of women from Québec clearly support previous findings of concentrations being considerably higher in North America compared with other parts of the world. The Québec levels do seem a little lower than those in the United States (Table 5).

In our group of Québec women, PBDE-47 is the dominating congener, and that also seems to be the case in the samples from the United States (Table 5). In European samples, however, PBDE-153 is often the dominating congener (Fängström et al. 2005; Johnson-Restrepo et al. 2005; Morland et al. 2005; Schecter et al. 2003). This difference in congener patterns between Europe and North America may be explained by differences in the use of penta-PBDE formulations, in which PBDE-47 is a main constituent. The European penta-PBDE technical mixture Bromkal 70-5DE was not used as extensively and has been banned for a longer period of time compared to the North American penta-PBDE mixture DE-71 (Birnbaum and Staskal 2004). Exposure to DE-71 is still occurring in North America; therefore, people are being exposed to PBDE-47 on a day-to-day basis. In Europe, exposure to the penta-PBDE formulation has declined and is probably rare nowadays. The PBDE congener pattern in plasma of people exposed several years ago to a penta-PBDE mixture but with little current exposure to this mixture is expected to shift in favor of the more persistent congeners. The estimated half-life (t_1/2_) of PBDE-153 (t_1/2_ = 6.5 years), a minor component of the penta-PBDE and octa-PBDE mixtures (La Guardia et al. 2006), is three times longer than that of PBDE-47 (t_1/2_ = 1.8 years); therefore, over time PBDE-153 should become the dominant one (Geyer et al. 2004; Hagmar et al. 2000; Thuresson et al. 2006). We (as most other authors) did not measure PBDE-209 in the plasma samples of participants. A deca-PBDE mixture, mainly composed of PBDE-209, has replaced the penta-PBDE mixture in several applications in Europe. PBDE-209 has been reported to be the major congener in human serum in one study (Inoue et al. 2006). Although it is unlikely that PBDE-209 would be a dominant congener in North American samples, it might be an important one in European samples, along with lower brominated congeners resulting from its biotransformation.

Considering that PCB levels are age and sex dependent, care should be exerted when comparing PCB-153 levels with those in other studies. Nevertheless, it seems clear from data in Table 5 that PCB-153 levels are lower in North America than in Europe. In a recent Canadian study, Tsuji et al. (2006) reported GM PCB-153 concentrations to be 50.2, 53.6, and 27.0 ng/g lipids in women from Fort Albany (Ontario), Kashechewan (Ontario), and Hamilton (Ontario), respectively. The first two groups were composed of First Nation women. Plasma concentrations measured in Québec City women are slightly lower than those measured in native women from Ontario but higher than those determined in non-native women from the same province.

Correlation analyses revealed that PBDE-153 was only moderately correlated to PBDE-47 (Figure 1D). We also observed that the contribution of PBDE-47 to ∑PBDEs increased with increasing concentrations of ∑PBDEs, whereas the reverse was true for the contribution of PBDE-153 to ∑PBDEs (Figure 2). These results again suggest that the sources of exposure to PBDE-153 might be different from those of the other PBDEs. Individuals with high PBDE plasma concentrations and a pattern of congeners dominated by PBDE-47 may have been directly exposed to the DE-71 mixture in which PBDE-47 and PBDE-99 are the dominating compounds. Inhalation and dermal contact with technical mixtures of PBDEs in commercial products may contribute substantially to exposure (Sjödin et al. 2004), and several studies in North America have reported dust and indoor exposure to be significant sources of PBDE exposure (Schecter et al. 2005a; Stapleton et al. 2005; Wu et al. 2007). Interestingly, some studies have reported higher plasma levels of PBDEs in children than in adults (Fischer et al. 2006; Thomsen et al. 2002), which may reflect the elevated dust exposure of toddlers (Jones-Otazo et al. 2005).

In individuals with lower PBDE plasma levels, the following lines of evidence indicate that exposure may originate in part from food consumption. We observed that the proportion of total PBDEs represented by PBDE-153 increases as total PBDE concentrations decrease. Similar to PCBs, weathering of PBDEs in the environment should lead to mixtures that are enriched in long-lived congeners such as PBDE-153 and lower in less persistent, lower-brominated congeners. The weak but statistically significant correlation observed between PCB-153 and PBDE-153 levels supports the interpretation of a common source of exposure to these compounds. Because exposure to PCBs is mainly through food consumption, this supports our contention that PBDE-153 exposure also occurs in part through food consumption. We did not conduct a complete dietary assessment of participants, but our questionnaire included questions on the consumption of a limited number of food items, including meat. We did not find any relation between meat consumption and plasma concentrations of PBDEs or PCBs. Hence, our results differ from those of Wu et al. (2007), who recently reported a positive association between meat consumption and PBDE concentrations in breast milk samples from 46 first-time mothers living in the greater Boston (Massachusetts) area. The consumption of fish has been linked to higher plasma PCB levels in Great Lakes fish consumers (Humphrey et al. 2000) and in postmenopausal U.S. women (Moysich et al. 2002; Wolff et al. 2005). It would have been interesting to examine relations of plasma PCBs and PBDEs to fish consumption in our study; unfortunately, we did not document fish consumption in our group of Québec women.

To our knowledge, no study conducted to date has found significant relations between PBDE concentrations in biological samples and age, BMI, parity, breast-feeding duration, or lifestyle habits. In contrast, we identified age and BMI as significant predictors of plasma PCB levels in our sample of Québec women. Similar associations had previously been reported in the two U.S. studies mentioned above (Moysich et al. 2002; Wolff et al. 2005). The lack of association noted between PBDE levels and age or BMI again supports the idea that the sources of exposure are different for PCBs and PBDEs. The lack of age factor might be explained by the relatively recent introduction of PBDEs, different exposure pathways, and the greater metabolism and elimination of PBDEs compared with PCBs (Johnson-Restrepo et al. 2005).

The final multiple linear regression model for plasma PCB-153 concentrations included age, current BMI, and BMI gain since 18 years of age as predictors and explained 34% of the variance (Table 4). Including BMI gain in the model increased the *R*^2^ value by 13% compared with the model containing only age and current BMI as independent variables (data not shown). Including BMI gain in the model also changed the direction of the association between current BMI and PCB-153 levels from negative to positive. Wolff et al. (2005) obtained a similar multivariate model that explained 30% of the variance in plasma PCB concentrations of 999 postmenopausal women on Long Island, New York. The major influence of BMI gain in our model could be explained by corresponding changes in the volume of the adipose tissue compartment, which is the major site of storage for PCBs and other lipophilic compounds. The greater the increase in the volume of the adipose tissue compartment, the greater the dilution of PCB residues and the lower their plasma concentration.

PBB-153 was detected in only 30% of the samples and was neither correlated to PBDEs nor to PCB-153. Levels of PBB-153 were also considerably lower than those of the PBDEs, as previously reported for the U.S. population (Sjödin et al. 2004).

One limitation of our study is the use of a convenience sample of postmenopausal women instead of a random population sample. It is likely that women recruited in breast cancer screening clinics are not representative of the general population of women on several characteristics. Compared with women from the general population, women in our study were more educated and smoked less than women from the general population. This would not however compromise the validity of our results, although they might be less amenable to generalization. Our study provides a first estimate of PBDE body burden in Canadian postmenopausal women. The major strengths of our study are its relatively large sample size and the extensive quality control–quality assurance procedures implemented for the analytical work.

In summary, the concentrations of organobromine compounds in plasma samples of Canadian women were slightly lower than those in the United States. However, the fact that the GM concentration of PBDE-47 is only 6 times lower than that of PCB-153 but the arithmetic means are comparable, clearly indicates the need to monitor PBDEs in human populations on a regular basis. The reason for the extreme levels determined in some women needs to be elucidated; there is also a need to identify the most important sources of human exposure to PBDEs in the general population.

## Figures and Tables

**Table 1:** Selected characteristics of the 110 postmenopausal women, Québec, Canada.

Characteristic	No. (%)	Mean ± SD	Range
Age (years)		58.3 ± 5.6	48 to 76
Weight (kg)		68.7 ± 14.0	44.8 to 133.0
Height (cm)		160 ± 5.2	146 to 172
BMI (kg/m^2^)		27.0 ± 5.4	17.2 to 51.3
BMI gain since 18 years of age (kg/m^2^)		6.4 ± 5.4	−14.7 to 28.0
No. of children		1.9 ± 1.4	0 to 5
Cumulative breast-feeding duration (weeks)		12.9 ± 26.5	0 to 124.3
Alcohol consumption (no. of drinks/day)		0.46 ± 0.56	0 to 3
Meat consumption (g/day)		59.7 ± 46.2	1.8 to 220.0
Smoking			
Current	8 (7.3)		
Ever	54 (49.1)		
Never	48 (43.6)		
Level of education			
Primary	9 (8.2)		
High school	26 (23.9)		
College or university	75 (68.2)		

**Table 2:** Lipid weight concentrations (ng/g lipids) of selected organobromine compounds in plasma samples from 110 postmenopausal women, Québec, Canada.

Compounds	Mean	GM	Range	Percent detected
PBDEs				
PBDE-47	39.0	8.10	0.81–1,780	100
PBDE-99	11.6	1.40	< 0.40–716	90
PBDE-100	6.79	1.10	< 0.40–366	83
PBDE-153	5.38	1.35	< 0.40–198	96
∑PBDEs	63.7	13.4	0.81–3,060	100
PBB-153	0.46	0.22	< 0.40–20.1	30

A value equal to one-half the LOD was substituted for non-detects to calculate mean values.

**Table 3:** Lipid weight concentrations (ng/g lipids) of PCB congeners in plasma samples from 109 post-menopausal women, Québec, Canada.

Congener	Mean	GM	Range	Percent detected
PCB-74	10.8	9.41	<3.80–50.5	97
PCB-99	9.14	6.66	<1.41–64.3	94
PCB-105	2.30	1.80	<1.00–13.4	69
PCB-118	13.1	11.2	3.81–69.9	100
PCB-138	25.9	22.7	5.45–101	100
PCB-146	4.72	4.01	1.55–33.8	100
PCB-153	47.1	41.7	14.4–177	100
PCB-156	5.36	4.77	2.15–24.7	100
PCB-163	17.1	14.5	5.39–124	100
PCB-167	1.60	1.30	< 0.96–8.52	66
PCB-170	12.9	11.4	5.57–65.2	100
PCB-172	1.62	1.30	< 0.72–11.5	64
PCB-177	1.76	1.46	< 0.80–8.68	72
PCB-178	2.29	1.80	< 0.94–19.6	81
PCB-180	40.4	34.7	1.39–206	100
PCB-183	3.09	2.71	<1.30–9.3	96
PCB-187	9.00	7.56	2.26–62.2	100
PCB-194	7.35	6.36	< 0.57–42.1	99
PCB-195	1.20	1.01	< 0.72–5.44	50
PCB-196	2.10	1.78	< 0.80–9.09	84
PCB-199	2.64	2.20	< 1.06–17.8	88
PCB-203	4.53	4.04	< 0.82–21.1	99
PCB-206	2.47	2.11	< 0.94–13.3	90
PCB-209	1.19	0.92	< 0.60–8.19	60
∑PCBs	229	204	93.1–1,010	

Only congeners detected in > 50% of the samples are listed. A value equal to one-half of the LOD was substituted for nondetects to calculate mean values. *n* = 109 (analytical problems were encountered with one sample).

**Table 4:** Multiple linear regression analysis of log-transformed PCB-153 concentrations (ng/g lipids) in plasma samples from 109 postmenopausal women, Québec, Canada.

	Unstandardized		Standardized	
	B	SE	β	*p*-Value
Age at interview (years)	0.007	0.003	0.202	0.012
Current BMI (kg/m^2^)	0.013	0.007	0.336	0.058
BMI gain since 18 years of age (kg/m^2^)	−0.031	0.007	−0.827	< 0.001

Model adjusted *R*^2^ = 0.335; *n* = 109 (analytical problems were encountered with one sample).

**Table 5:** Comparison of PBDE and PCB-153 levels in the present study with concentrations from other studies conducted elsewhere in the world.

Reference	PBDE-47	PBDE-99	PBDE-100	PBDE-153	PCB-153	Medium	Age	Sex	Country	No.
Present study	8.1	1.4	1.1	1.4	41.7^a^	Plasma	48–76	Female	Canada	110
Schecter et al. 2005b	12.8	3.2	2.6	3.6		Blood	22–91	Both	United States	39
Sjödin et al. 2004	34	11	5.9	7.3	35	Serum	?	Both	United States	Pool
Johnson-Restrepo et al. 2005	29.3	10.3	12.0	< 1	35.2^b^	Adipose tissue	18–51	Both	United States	52
Weiss et al. 2006	0.91	0.20	0.29	1.1	260	Serum	52–81	Female	Sweden	53
Thomas et al. 2006	0.82	< 0.16	0.76	1.7	41	Serum	22–80	Both	United Kingdom	154
Fängström et al. 2005	1.3	0.33	0.51	1.0	430	Serum	?	Female	Faroes	57
Naert et al. 2006	0.88	0.47	0.72	2.40	274	Adipose tissue	19–84	Both	Belgium	53

All levels are either GMs or medians and are expressed on a lipid basis (ng/g lipids).

a *n* = 109.

b Sum of hexa-chlorinated congeners.
